# Supplementary material for: Feasibility of estimating cardiac indices using cardiac surgery anesthesia records in a multicenter cohort
Source: J Clin Monit Comput. 2025 Dec 24;40(3):635–43. doi: 10.1007/s10877-025-01400-0 (PMC13110883; doi:10.1007/s10877-025-01400-0)

**Online Resource 1**. Supplemental Tables and Figures

**Supplemental Table 1.** Number of cases and primary anesthesia attendings by study hospital. Participating health systems are Brigham and Women’s Hospital, Boston, Massachusetts; Massachusetts General Hospital, Boston, Massachusetts; Oregon Health and Science University, Portland, Oregon; University of Michigan Health System, Ann Arbor, Michigan; University of Washington Medical Center, Seattle, Washington; Washington University of St. Louis School of Medicine, St. Louis, Missouri; Yale New Haven Hospital, New Haven, Connecticut.

| Hospital | Pre-CPB Patients  n (%) | Post-CPB Patients  n (%) | Pre-CPB Primary Anesthesia Attending  n (%) | Post-CPB Primary Anesthesia Attending  n (%) |
| --- | --- | --- | --- | --- |
| A | 1,219 (59) | 3,919 (70) | 35 (22) | 36 (18) |
| B | 598 (29) | 613 (11) | 45 (28) | 46 (23) |
| C | 73 (4) | 496 (9) | 29 (18) | 38 (19) |
| D | 105 (5) | 193 (3) | 22 (14) | 23 (11) |
| E | 22 (1) | 271 (5) | 10 (6) | 22 (11) |
| F | 33 (2) | 45 (1) | 15 (9) | 16 (8) |
| G | 4 (0) | 81 (1) | 3 (22) | 22 (11) |

**Supplemental Table 2.** Goodness-of-fit (GOF) statistics for the null, simple, and full models. Null model contains only hospital and primary anesthesiologist levels. Simple model contains these levels and age, gender, and race as covariates. Full model contains these levels and covariates in addition to procedure type (aortic, bypass, valve), presence of cardiac shock, central venous pressure (CVP), oxygen saturation, and vasoactive-inotropic score (VIS). AIC = Akaike Information Criterion; AICC = corrected Akaike Information Criterion; BIC = Bayesian Information Criterion.

| Pre-Cardiopulmonary Bypass (CPB) | |  |  |  |  |  |  |  |  |  |  |  |
| --- | --- | --- | --- | --- | --- | --- | --- | --- | --- | --- | --- | --- |
|  | MAP | | | Windkessel | | | Liljestrand and Zander | | | Herd | | |
| Goodness of fit | Null | Simple | Full | Null | Simple | Full | Null | Simple | Full | Null | Simple | Full |
| -2 Res Log Likelihood | 11505.428 | 11183.809 | 11152.123 | 11505.428 | 10983.961 | 10935.986 | 11505.428 | 10855.021 | 10812.793 | 11505.428 | 10981.764 | 10933.789 |
| AIC | 11511.428 | 11189.809 | 11158.123 | 11511.428 | 10989.961 | 10941.986 | 11511.428 | 10861.021 | 10818.793 | 11511.428 | 10987.764 | 10939.789 |
| AICC | 11511.433 | 11189.813 | 11158.128 | 11511.433 | 10989.966 | 10941.99 | 11511.433 | 10861.026 | 10818.797 | 11511.433 | 10987.769 | 10939.793 |
| BIC | 11510.804 | 11189.184 | 11157.499 | 11510.804 | 10989.337 | 10941.361 | 11510.804 | 10860.396 | 10818.168 | 11510.804 | 10987.139 | 10939.164 |
|  |  |  |  |  |  |  |  |  |  |  |  |  |
| Post-Cardiopulmonary Bypass (CPB) | |  |  |  |  |  |  |  |  |  |  |  |
|  | MAP | | | Windkessel | | | Liljestrand and Zander | | | Herd | | |
| Goodness of fit | Null | Simple | Full | Null | Simple | Full | Null | Simple | Full | Null | Simple | Full |
| -2 Res Log Likelihood | 4103.4621 | 4046.6867 | 4063.8316 | 4103.4621 | 3958.573 | 3968.177 | 4103.4621 | 3917.8643 | 3925.6813 | 4103.4621 | 3956.3758 | 3965.9797 |
| AIC | 4109.4621 | 4052.6867 | 4069.8316 | 4109.4621 | 3964.573 | 3974.177 | 4109.4621 | 3923.8643 | 3931.6813 | 4109.4621 | 3962.3758 | 3971.9797 |
| AICC | 4109.4738 | 4052.6985 | 4069.8434 | 4109.4738 | 3964.5848 | 3974.1887 | 4109.4738 | 3923.876 | 3931.6931 | 4109.4738 | 3962.3876 | 3971.9915 |
| BIC | 4109.2999 | 4052.5244 | 4069.6693 | 4109.2999 | 3964.4108 | 3974.0147 | 4109.2999 | 3923.702 | 3931.519 | 4109.2999 | 3962.2135 | 3971.8175 |

# **Supplemental Table 3.** Correlation matrix for all cardiac index estimators. MAP = mean arterial pressure.

|  | MAP | Windkessel | Herd | Liljestrand and Zander |
| --- | --- | --- | --- | --- |
| MAP | 1.00 | 0.49 | 0.49 | 0.18 |
| Windkessel | 0.49 | 1.00 | 1.00 | 0.89 |
| Herd | 0.49 | 1.00 | 1.00 | 0.89 |
| Liljestrand and Zander | 0.18 | 0.89 | 0.89 | 1.00 |

**Supplemental Table 4.** Attributable variance statistics hospital and primary anesthesiologist. Null model contains only hospital and primary anesthesia attending levels. ICC = Intraclass Correlation Coefficient; VPC = Variance Partition Coefficient.

| Pre-Cardiopulmonary Bypass (CPB) | |  |  |  |  |  |  |  |  |  |
| --- | --- | --- | --- | --- | --- | --- | --- | --- | --- | --- |
|  | Null Model | | Full model | | | | | | | |
|  |  |  | MAP | | Windkessel | | Liljestrand and Zander | | Herd | |
| Level | ICC | VPC | ICC | VPC | ICC | VPC | ICC | VPC | ICC | VPC |
| Primary Anesthesiologist | 0.513 | 9.82 | 0.316 | 10.01 | 0.069 | 9.12 | 0.069 | 9.12 | 0.069 | 9.12 |
| Hospital | 9.307 | 9.31 | 9.691 | 9.69 | 9.047 | 9.05 | 9.047 | 9.05 | 9.047 | 9.05 |
|  |  |  |  |  |  |  |  |  |  |  |
| Post-Cardiopulmonary Bypass (CPB) | |  |  |  |  |  |  |  |  |  |
|  | Null Model | | Full model | | | | | | | |
|  |  |  | MAP | | Windkessel | | Liljestrand and Zander | | Herd | |
| Level | ICC | VPC | ICC | VPC | ICC | VPC | ICC | VPC | ICC | VPC |
| Primary Anesthesiologist | 2.212 | 11.38 | 2.363 | 11.05 | 2.180 | 11.22 | 2.421 | 11.18 | 2.180 | 11.22 |
| Hospital | 9.171 | 9.17 | 8.686 | 8.69 | 9.044 | 9.04 | 8.758 | 8.76 | 9.044 | 9.04 |

# **Supplemental Table 5.** Confusion matrices for all cardiac index estimators in discriminating low CI (<2.2 L/min/m2) from normal or high CI (>=2.2 L/min/m2) compared to reference standard CI values from thermodilution. CI = cardiac index; CPB = cardiopulmonary bypass; MAP = mean arterial pressure.

|  | **Pre-CPB** | | | | | **Post-CPB** | | | | |
| --- | --- | --- | --- | --- | --- | --- | --- | --- | --- | --- |
| MAP |  |  | Predicted | |  |  |  | Predicted | |  |
|  |  |  | *Normal* | *Low* |  |  |  | *Normal* | *Low* |  |
|  | Actual | *Normal* | 608 | 317 |  | Actual | *Normal* | 3143 | 398 |  |
|  |  | *Low* | 484 | 645 |  |  | *Low* | 1583 | 494 |  |
|  |  |  |  |  |  |  |  |  |  |  |
| Windkessel |  |  | Predicted | |  |  |  | Predicted | |  |
|  |  |  | *Normal* | *Low* |  |  |  | *Normal* | *Low* |  |
|  | Actual | *Normal* | 605 | 320 |  | Actual | *Normal* | 3119 | 422 |  |
|  |  | *Low* | 445 | 684 |  |  | *Low* | 1486 | 591 |  |
|  |  |  |  |  |  |  |  |  |  |  |
| Herd |  |  | Predicted | |  |  |  | Predicted | |  |
|  |  |  | *Normal* | *Low* |  |  |  | *Normal* | *Low* |  |
|  | Actual | *Normal* | 605 | 320 |  | Actual | *Normal* | 3119 | 422 |  |
|  |  | *Low* | 445 | 684 |  |  | *Low* | 1486 | 591 |  |
|  |  |  |  |  |  |  |  |  |  |  |
| Liljestrand and Zander |  |  | Predicted | |  |  |  | Predicted | |  |
|  |  |  | *Normal* | *Low* |  |  |  | *Normal* | *Low* |  |
|  | Actual | *Normal* | 599 | 326 |  | Actual | *Normal* | 3094 | 447 |  |
|  |  | *Low* | 430 | 699 |  |  | *Low* | 1441 | 636 |  |
|  |  |  |  |  |  |  |  |  |  |  |

#
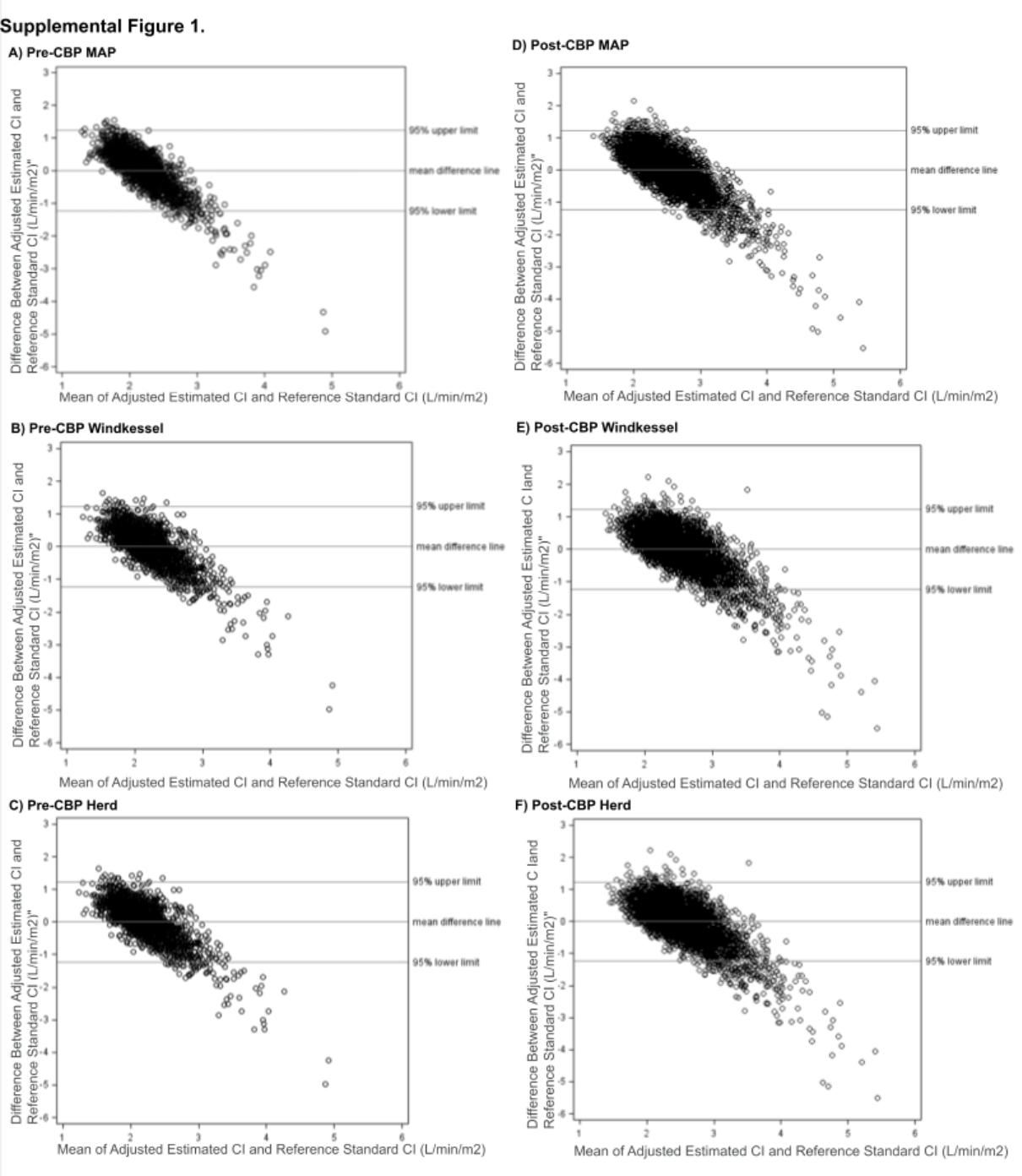
**Supplemental Figure 1.** Bland-Altman plots for the MAP, Windkessel, and Herd estimators pre- and post-CPB. CI = cardiac index; CPB = cardiopulmonary bypass; MAP = mean arterial pressure.

# **Supplemental Figure 2.** Scatter plots for the CI estimators pre- and post-CPB with regression line (blue) 95% confidence limits (light blue). CI = cardiac index; CPB = cardiopulmonary bypass; MAP = mean arterial pressure.

#
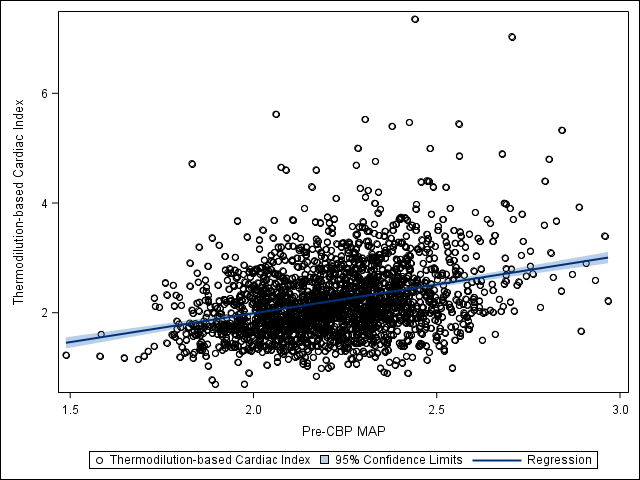

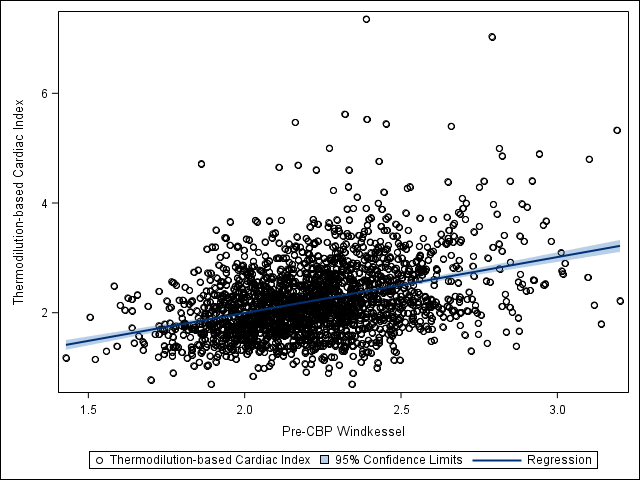


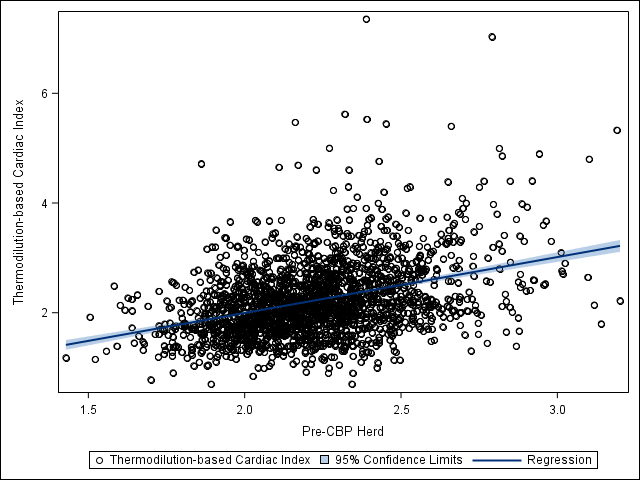

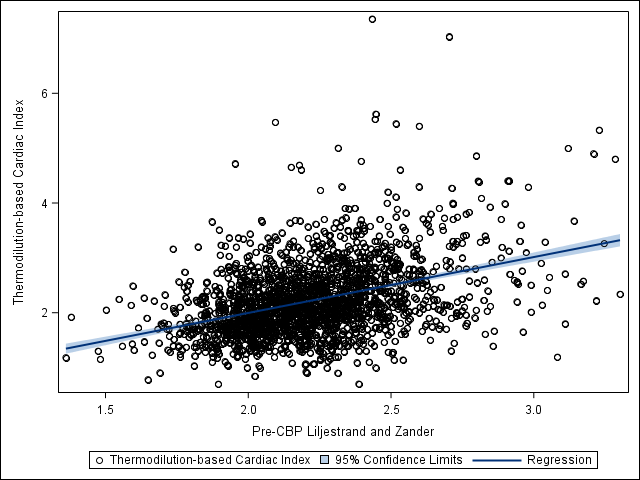


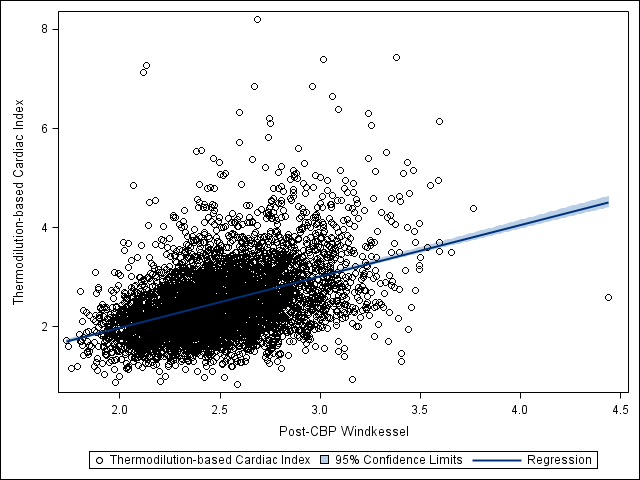

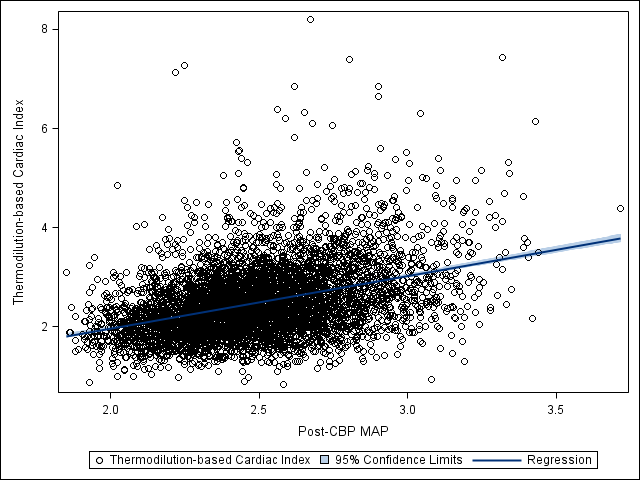


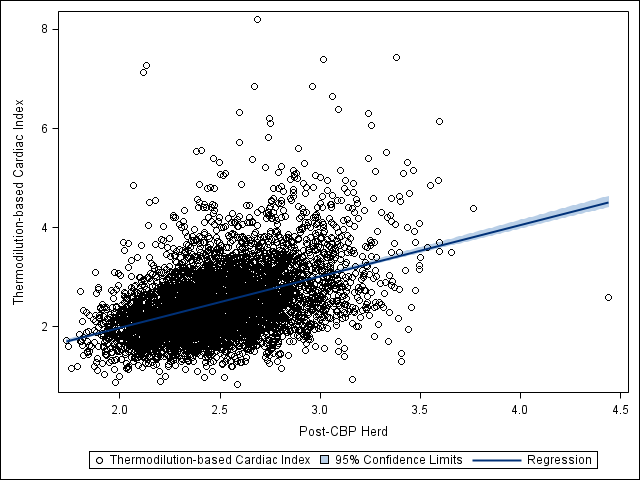

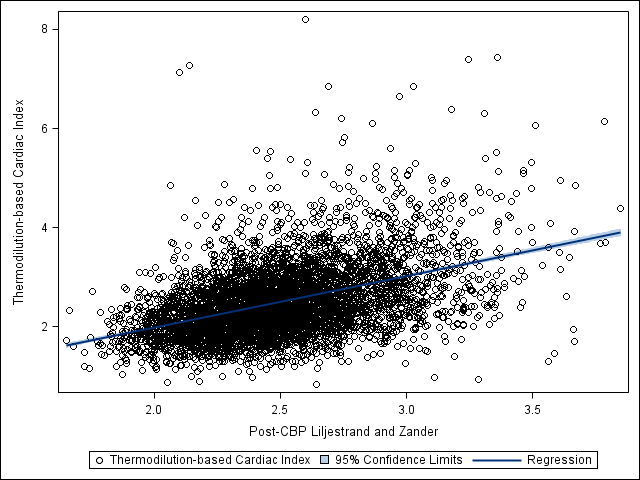

Supplement: Supplementary file 2 — Supplementary Material 2 [file 10877_2025_1400_MOESM2_ESM.docx]
